# Supplementary material for: Are carbon emissions trading and green financial instruments synergistic? -Comprehensive quantitative research based on content analysis
Source: PLoS One. 2024 Mar 7;19(3):e0298601. doi: 10.1371/journal.pone.0298601 (PMC10919601; doi:10.1371/journal.pone.0298601)
Supplement: S1 Data — (ZIP) [file pone.0298601.s001.zip › Data/地级市Z_F工作报告.zip!/╜¡╬≈/╛┼╜¡╩╨/└┤╫╘╣1⁄2╓┌║┼í╛┬φ┐╦╩2╛▌═°í┐_2010.docx]

各位代表：

现在，我代表市人民政府，向大会报告工作，请予审议，并请各位市政协委员和列席会议的同志提出宝贵意见。

　　一、2009年工作回顾

　　2009年，是新世纪以来我国经济发展最为困难的一年，也是我市实施赶超战略的第一年。在省委、省政府和市委的正确领导下，我们坚持以科学发展观为统领，按照“三保一弘扬”的要求，深入贯彻市委九届七次全会的决策部署，努力克服国际金融危机带来的不利影响，紧紧依靠全市人民，化危机为赶超的契机，变挑战为发展的机遇，一手抓发展、一手托民生，扎实工作，奋起赶超，全面完成了市十三届人大四次会议确定的各项任务，预计实现生产总值830亿元，增长14.5%，各项经济指标在全省排位全部摆脱后三进入中三，部分指标达到前三，实现了“三年翻番、五年进位”的头年初胜，首战告捷。

——固定资产投资实现赶超式进位。完成全社会固定资产投资659亿元，增长43.9%，增幅由上年全省第十一位进到第一位；城镇固定资产投资647亿元，增长46.1%，增幅列全省第二；工业固定资产投资434亿元，增长46%，占固投总量65.9%。

——工业经济实现赶超式进位。规模以上工业主营业务收入突破1000亿元、利税总额突破100亿元、主营业务收入过亿元企业突破200户；规模以上工业增加值、主营业务收入、利税总额全部实现收复性进位，总量重归全省第二；工业经济效益综合指数216%，同比提高43个百分点，提高值居全省首位。中石化九江分公司为市本级纳税首次突破10亿元。

——财政收入实现赶超式进位。财政总收入实现86.19亿元，增长30.3%，总量位居全省第三，净增20亿元，相当于前两年增量的总和，增速由上年的全省第十位进到第二位；完成地方一般预算收入50.5亿元，增长31.4%；税收占财政收入的比重达85.9%，比上年提高0.9个百分点；财政总收入占GDP比重达10.4%，比上年提高0.8个百分点。

——城市建设实现历史性突破。城建力度空前加大，城建项目高效推进，城市框架拓展一倍。8个月内先后启动三批次十大重点工程，中心城区基础设施和公益设施投入规模突破50亿元，超过前五年的总和，经营性土地出让收入超过前四年的总和，实现了城市建设投资总额、项目个数、建设速度、融资规模、土地收益、规费收缴 “六个超历史”。中心城区“从甘棠湖、南湖时代走向八里湖时代”迈出了坚实的步伐。

——重大项目实现历史性突破。全年新引进、开工、竣工投产10亿元以上的产业和基础设施项目46个。彭泽核电、十大电网、昌九城际等一大批能源交通项目密集建设；星火有机硅一体化、江铜铅锌冶炼等一大批产业龙头项目快速推进；九江石化800-1000万吨炼化一体化、九江电厂“上大压小”等一批技改扩能项目取得重大突破，不仅有效拉动了当前经济发展，更为今后的发展奠定了坚实基础。

——改革攻坚实现历史性突破。国有企业改革在全省率先突破，采取“建立政府融资平台、打捆企业资产融资、全面置换职工身份、处置资产还债重组”的办法，全市126户国有工业企业基本完成改制，安置职工7.7万人，安置率达95.9%；市属34户国有工业企业全面完成职工身份置换，近3万职工“三金”得到落实。市县两级土地利用总体规划修编纲要通过审批，积极争抢建设用地指标3.5万亩，居全省首位，保障了项目用地需求。城建规费实行联审联批、统一收缴，全年净增1.7亿元，增长245%。对群众反映强烈的城区“两证”发放等历史遗留问题，依法进行妥善处理，为2万市民补发房屋产权证和土地使用证。鄱阳湖采砂禁采一年之后，以统一经营管理的方式进行保护性开采，实现了生态效益、社会效益、资源效益的高度统一。

——扩大开放成效显著。实际利用外资5.2亿美元，增长19.3%，总量全省第三，增幅由上年的十一位达到全省首位；新批外资项目172个，全省第一。全年引进省外5000万元以上工业项目129个，实际进资138.6亿元，省外亿元以上工业项目35个，实际进资70.8亿元，均列全省前三位；外贸出口总额4.6亿美元，增长75.4%，增幅由上年第九位前移至第二位。民营经济加快发展，全市民营企业完成税收43.2亿元，首次占到财政“半壁江山”。

——筹资融资成效显著。充分发挥投融资平台功能，广泛开展政银企对接,“想建设无钱搞建设、想干事无钱干成事”的难题得到有效破解。全年市本级累计融资67.6亿元，是上年的5倍；年末全市金融机构本外币各项贷款余额514亿元，增长45.7%，比年初净增161.3亿元，是上年的两倍多，增量和增幅居全省前列；12亿元城投债券首获发行；九江银行资产总规模突破200亿，一年内实现翻番；新增招商银行、南昌银行、江南证券等六家金融机构入驻九江，共青恒信小额贷款公司纳入全省首批试点；九江被评为“中国最具影响力金融生态示范城市”。

——效能建设成效显著。建立全省首家“民声直通车” 自动化综合平台，受理咨询、建议和诉求5187件，办结率达99.3%。严肃整治“官场”和市场，成效明显。加大财政投资评审监管力度，核减预算资金近2亿元。行政许可项目、投资项目涉及的审批事项、审批平均时限全部缩减35%以上，超额完成“三个缩减30%”的任务。获得2009年度全省机关效能年活动绩效考核第三名。

——县域经济加快赶超。在强工兴城的同时，“三农”基础地位继续得到巩固。粮食总产153.5万吨，实现“六连增”；棉花总产9.1万吨，湖口县武山镇百亩示范基地亩产籽棉1014斤，创造了长江、黄河流域两大棉区的大面积单产新纪录；油菜总产全省第一，增长33.6%，创历史新高。各县（市、区）以“两区”为主战场，以项目为主抓手，争先创优，奋起赶超，园区投入全面加大，县城面貌显著改观，发展步伐明显加快。全年完成县级财政总收入65亿元，对全市财政的贡献率达75.5%；实现财政收入县县过2亿，7个县过5亿，3个县过6亿；六个县（区）财政总收入增幅超40%，湖口县达到58%。

--人民生活加快改善。93件为民实事基本完成，省级文明城市创建工作全面推进，“两声三乱”和城乡环境综合整治成效明显，人民群众得到更多实惠，生活水平进一步提高，生活环境进一步改善，幸福指数进一步提升。城镇居民人均可支配收入14203元、增长10.2%，职工年平均工资收入20466元、增长15%，农民人均纯收入4819元，增幅位居全省第一。全市居民储蓄存款488.6亿元，比年初增加102.2亿元，增幅高出全省4.3个百分点。居民消费价格指数与上年持平，完成社会消费品零售总额240.5亿元，增长19%。

--社会事业加快发展。在经济加快发展的同时，一批重大公共设施项目加快推进，各项社会事业协调发展。特别是在改革和建设中，群众利益得到较好维护，民求民盼得到较好解决，社会保持和谐稳定，人民群众安居乐业，全市上下呈现一幅国泰民安的景象。在全省社会治安群众安全感测评中名列第三。

一年来，在市委的坚强领导下，全市上下紧扣赶超目标，实施赶超战略，投身赶超实践，加快赶超步伐，主要做了以下工作：

（一）紧紧抓住工业总量不大、经济实力不强的矛盾，强攻工业加快赶超。主要抓了五个方面的工作：一是着力帮企业、保运行。落实“两降三促一优先”政策，开展帮办证照、帮建设、帮招工、帮融资“四帮”服务，分期分批帮助企业解决难题；积极组织政银企对接，为30户民营企业融资60亿元，为409户企业提供11.3亿元贷款担保，为10户困难企业减免缓交税费2500多万元，兑现增值税转型等政策，为企业减、免、抵税收10亿元以上；设立“企业之声”平台，办结企业诉求195件，帮助企业渡过难关，更快发展。全年净增规模以上企业116户，工业用电量达45.2亿度、增长20.7%，增幅高于全国、全省平均水平。二是着力攻项目、强龙头。以项目建设为总抓手，盯住大企业，主攻大项目，广泛开展央企对接，推进一批重大企业追加投资、技改扩能、退城进园、做大做强。大力招商引资，一批大项目、大企业落户九江。全年新开工亿元以上工业项目88个，其中10亿元以上项目9个；新竣工投产亿元以上工业项目24个。三是着力兴产业、蓄优势。依托沿长江、沿昌九和产业基础优势，延展产业链条，培育产业集群。石油化工、冶金建材、纺织服装、电力能源、汽车船舶等优势产业不断壮大，新型能源、电子信息等新兴产业加快成长，全市已形成3个国家级、10个省级产业基地，多点支撑、多极发展渐成体系。四是着力攻园区、建平台。按一级准政府的体制加强工业园区管理，投入23.3亿元，加强园区基础设施建设，园区开发面积达55.4平方公里，增长44%；园区新增落户企业118户，规模以上工业总量占全市的77.4%，对全市工业增长贡献率达93.3%；园区经济在全省的位次前移，湖口工业园主营业务收入突破100亿元，成为全市第二个超百亿园区，九江经济开发区进入全省“十强园区”，申报国家级开发区取得新进展。五是着力抓减排、降能耗。积极推进污染减排、污染治理工程，全市万元生产总值能耗下降4.8%，化学需氧量排放量下降3.04%，二氧化硫排放量下降16.6%，均控制在省定指标之内。

（二）牢牢把握兴城改变面貌、城建撬动发展的杠杆，振兴城市加快赶超。主要抓了五个方面的工作：一是全力以赴建设新城。按照市委构建“大九江”的要求，完善了城市总体规划，完成了八里湖新区控制性详规修编，启动编制八里湖新区四个重点区域的修建性详规。以基础设施和公共服务设施为重点，全力推进项目建设，着力打造八里湖新区，拉开城市走向“八里湖时代”的序幕。长虹西大道、八里湖环湖二路建成通车；十里河（濂溪河）综合整治、八里湖大道、环湖一路、濂溪大道、体育中心、庐山旅游直升机等项目主体工程基本完工；八里湖新区学校、新区医院、兴城大道及体育中心配套路网、赛城湖公园、八里湖跨湖大桥引道、第二生活垃圾处理场等工程开工建设。二是统筹兼顾提升老城。完成大中路西段、庾亮南路历史文化街区修复规划编制，整修和完善了“两湖”周边设施；实施了扬子巷、花园畈等片区棚户区改造；开工建设浔阳东路地下人防工程；新建和改建一批农贸市场、公厕和垃圾中转台。三是着力破解资金难题。更新城市经营理念，优化城市经营模式，高度垄断土地一级市场，变“卖地筹资”为“储地融资”；变“先卖地后做环境”为“先做环境后卖地”，加大闲置用地清理力度，清收土地8000亩，新增土地融资35.6亿元，实现土地出让收入34.7亿元，保障了城建资金需求，探索出“以城养城、以城建城”的新路子。四是扎实有效管理城市。进一步完善城市管理长效机制，加大执法力度，先后开展“两声三乱”、占道亭棚、户外广告等综合整治，积极创建省级文明城市，城市管理水平不断提升，荣获“中国最佳管理城市”称号。五是加快推进城镇建设。各县（市、区）把兴城摆在更加突出的位置，大力推进城镇建设，县城框架加快拓展，县城功能不断完善，县城面貌发生明显变化。九江县港口街镇、庐山区新港镇等一批重点集镇加快建设。

（三）全面树立项目主导发展、投资决定增长的理念，主攻项目加快赶超。主要抓了三个方面的工作：一是积极向上争取并配合推进一批能源、交通重大基础设施项目。抓住国家扩大内需、刺激增长的政策机遇，积极争资争项，全力配合推进。长岭、大岭风电等项目建成投运；武九铁路改造、九瑞高速、彭湖高速、长江二桥、彭泽核电、西气东输、柯龙线改造等项目有序推进；32个重大项目列为省重点工程，占全省的1/5，全年完成投资118亿元。二是积极对外招引并强力推进一批工业产业项目。抓住产业转移机遇，精心组织香港、北京等地集中招商活动，积极参与世界低碳经济大会、赣台经贸合作研讨会、中国“五会”等招商活动，开放对接成效明显。投资50亿元的湖口粒状合金等项目签约；投资80亿元的星火有机硅、投资50亿元的江铜铅锌冶炼等项目开工建设；九钢150万吨板材、巨石30万吨玻纤、诺贝尔陶瓷、龙达化纤一期、旭阳雷迪多晶硅、塑丽龙纺织等项目竣工投产。三是积极加大投入并加快推进一批民生和社会事业项目。在推进十里河综合整治、八里湖新区学校、新区医院等重大项目的同时，启动建设市儿童福利院，加快推进市特教学校康复楼、市科技中专新校区、市中医院南院、市妇幼保健院大楼和县区老年公寓建设，建成浔阳区三里小学、庐山区怡康小学。

（四）切实巩固“三农”基础地位不动摇，夯实基础加快赶超。主要抓了四个方面的工作：一是推进农业产业建设。建立粮棉油万亩示范片26个，培育种养大户5200户。新增标准化生产基地94个，标准化种养面积达到178.8万亩。新增省级以上农产品品牌9个、有机绿色食品品牌47个。新增农民专业合作社350家，入社农民2.6万人。二是推进农业项目建设。引进3000万元以上农业项目87个，实际进资17.7亿元。全市233家规模以上农业企业实现销售收入160亿元，增长13%。三是推进农村基础建设。建成1510公里农村水泥路，全市行政村通畅率97%，通达率100%。推进改渡建桥45座，完成50座小(二)型以上病险水库除险加固，改造中低产田10万亩。建设1135个新村点，新农村建设继续保持全省先进水平。四是推进农村社会事业建设。全面落实强农惠农政策，发放农业各项补贴2.9亿元；补贴汽车、摩托车、家电下乡17万台（件），资金8200万元；新建和改造1000家“万村千乡市场工程”连锁农家店；整村推进357个重点村扶贫开发，完成深山区移民搬迁4799人；发放库区移民后期扶持补助2亿元；农场税费改革基本完成，林权配套改革深入推进。扶持返乡农民工就业创业，近50万春节期间返乡农民工实现再就业，3700人实现了自主创业。

（五）十分注重把潜在的优势转化为现实的发展，挖潜扬优加快赶超。主要抓了四个方面的工作：一是注重发挥旅游资源优势。旅游各项经济指标的总量均列全省第一，全年接待境内外游客突破1500万人次，增长16%，旅游总收入突破100亿元，增长20％。旅游基础设施建设取得重大突破，全省首条旅游高速（永武高速）开工建设，中信集团投资 120亿元的庐山西海开发建设项目签约。设立旅游促销基金，举办首届中国庐山世界名山大会，九江被评为“最具国际影响力旅游城市”。二是注重发挥沿江优势。城东、城西等沿江六大板块的临港产业加快发展，全年新开工项目37个，新投产项目19个；启动建设九码快速通道，积极推进九江电子口岸纳入全省建设重点，全力争取城西港区第七类废弃物转关获得批准，成功实现九江港与南昌港“两港两航”战略合作，集装箱首破10万标箱，九江港区域性中转港的地位开始凸显。三是注重发挥鄱阳湖生态优势。策应鄱阳湖生态经济区建设，启动建设环湖景观林带，治理水土流失40.6万亩，11个县级污水处理厂基本建成投运，农村生活垃圾无害化处理保持全省领先；积极发展新型清洁能源，全市风电总装机达8.4万千瓦，华航太阳能、中辉特光伏等一批新型能源项目加快推进。四是注重发挥共青城独特优势。进一步理顺管理体制，给足发展条件，加大支持力度，推进共青城与德安相向发展，共青城全国青年创业基地成功授牌，园区设施逐步完善，金淞电器、启维光伏、中芬数字生态城等一批重大项目落户开工。固定资产投资等主要经济指标高位增长，实际利用外资实现100%以上增幅，注意力经济开始显现效益。

（六）始终坚持一手抓发展、一手托民生的原则，发展为民加快赶超。主要抓了三个方面的工作：一是提升就业创业水平。新增城镇就业7.9万人，新增转移农村劳动力10.9万人，新增小额担保贷款4.4亿元，新增个体户2.6万户、私营企业2800户。免费培训9.2万农村劳动力，免费进行技能和创业培训6.6万人，扶助就业创业工作居全省首位。二是提升社会救助水平。城乡低保对象补助标准月人均补差分别达到180.3元、78.8元。农村五保户供养标准年人均提高60元。上世纪六十年代精简下放老职工救济金城乡月人均补助分别达到200元、160元，城镇“三无”特困群众供养标准月人均提高到300元。救助家庭困难学生4.2万人。对因突发性事件造成生活困难的群众实行临时救助。建立因自然灾害死亡人员抚慰金制度。为2.6万名残疾人免费提供康复服务。推进“光明·微笑”工程，完成白内障复明手术1.2万例、唇腭裂修复手术623例。三是提升各项保障水平。全市新建廉租房5300套、26.5万平方米，为1.4万户发放租赁补贴；中心城区新建经济适用房898套、为700户发放货币补贴；完成农村危房改造1476户。城镇职工养老保险参保人数达77.9万人，工伤保险参保人数达86.6万人，失业保险参保人数达34.3万人，被征地农民养老保险新增参保人数9.1万人，城镇职工基本医疗保险参保人数达65.1万人；在校大学生全部纳入城镇基本医疗保险范围，新型农村合作医疗参合农民达到331.3万人，统筹基金使用率达98.8%。大病医疗救助水平进一步提高。改扩建42所农村敬老院和3所福利中心；开展了居家养老试点工作。对离任老村支书、村主任给予生活补贴，对“三院”中符合条件的工作人员以及居委会工作人员给予工作补贴。

（七）大力促进各项社会事业全面发展，统筹协调加快赶超。主要抓了三个方面的工作：一是大力促进教、科、文、卫、体等事业加快发展。义务教育入学率、巩固率进一步提高，高中教育普及率仍居全省第一，新增省级重点中学8所，新扩建农村中小学校舍8万平方米；高等教育加快发展，九江学院在柬埔寨创设孔子学院，九江职业大学、九江职业技术学院、江西财经职业技术学院教育教学质量不断提升。争取省级以上科技计划项目62项，新增省级高新技术企业3家，申请专利400件、授权180件。推进医药卫生体制改革，没有发生重大疫情的暴发流行和死亡病例；新增4所医院获“三甲”称号。人口自然增长率控制在7.83‰。广播电视、新闻出版事业稳步发展，文物保护不断加强，新建农家书屋228家。全民健身和竞技体育进一步发展。推进人才强市战略，启动建设全省首家留学人员创业园。九江军转安置工作获国务院表彰。二是全力维护社会和谐稳定。全面推进安全生产“三项活动”，各类伤亡事故和死亡人数分别下降15.4%和7.6%，安全生产总体形势保持稳定。坚持领导定期接访、干部下访、矛盾排查调处制度，开展“信访积案化解年”活动，信访工作得到加强。强化治安防控体系，加强110快速反应机制建设，加大巡逻执勤力度，开展“打黑除恶治霸”专项行动，做好法律援助工作，保持了社会和谐稳定。三是统筹推进统计、审计、编制、人防、地震、气象、水文、海关、检验检疫、质监、信息化、应急管理、民族宗教、烟草专卖、档案、地方志、民兵预备役、工会、共青团、妇女儿童、老龄、残疾人、红十字和慈善等事业协调发展。

（八）以学习实践科学发展观为动力加强政府自身建设，提高效能加快赶超。主要抓了五个方面的工作：一是强化责任意识。始终坚持对党负责、为人民服务，以是否有利于事业发展、是否有利于人民幸福来衡量工作的成效，在最大的范围倾听民声，尽最大的力量改善民生，尽最大的努力解除民忧。自觉接受人大依法监督和政协民主监督，认真办理人大代表建议 270件、政协提案442件，办结率均达100%。二是强化团队效能。认真贯彻民主集中制，有效配置政府工作力量，充分放手放权，实行权责统一，分工到人，分管到位，各尽其责，各用其力，形成了分管市长前线打主攻、常务市长一线作协调、市长全线担责作保障的“三条线”工作格局，政府班子的积极性、创造性和协调性有效提高。三是强化推进机制。为确保各项工作落实到位，扎实有效，年初将政府主要工作分解成“六张作业单”，按职能分工，全方位、全战线推进：即省对市县的考核情况，市政府工作报告任务完成情况，重点工程和项目推进情况，93件民生实事完成情况，人大代表建议和政协提案、市政府“民声直通车”和“企业之声”等诉求办理情况，部门招商引资和争先进位情况。实行一季一调度，半年一考核，年终交总帐，形成了高效的台帐式推进机制。四是强化服务举措。按照“有请必示，有求必应，有难必帮，有急必救”的原则，采取强化考核、下放权力、理顺体制、现场办公等措施，激励县区争先创优，增强县区发展活力，帮助县区破解难题，服务县区加快发展。五是强化廉政建设。全面落实党风廉政责任制，完善行政监督体系，推进“廉政阳光工程”和“四中心一平台”建设，推行财政票据电子化管理和公务卡制度改革试点，开展“小金库”、中介机构、行业协会等专项治理工作，促进公共资源阳光交易、重大事项阳光决策、公共财政阳光运行、公共权力阳光监督。树立过紧日子思想，实现了公务用车、会议经费、公务接待费及党政机关出国（境）经费“零增长”。

各位代表，过去的一年，既是我市经济逆势上扬、开创新局的一年，也是全市上下攻坚克难、合力合成的一年。一年来，我们以科学发展观为指导，适应发展的需求，策应上级的要求，顺应群众的盼求，作出了强工兴城的新部署，营造了共识共为的好氛围，迈出了奋起赶超的新步伐，开启了腾飞向上的好势头。成绩的取得，是省委、省政府和市委坚强领导的结果，是市人大、市政协监督支持的结果，是全市人民和各界人士团结奋斗的结果。在此，我代表市人民政府，向辛勤劳动在各个领域的全市人民，向大力支持政府工作的各位人大代表、政协委员，向离退休老同志，向各民主党派、工商联、各人民团体和社会各界人士，向驻浔部队和武警官兵，向中央和省驻浔各单位，向所有关心、支持、参与九江建设和发展的各界朋友、各位纳税人，表示衷心感谢并致以崇高敬意！

回顾一年来的工作，我们感到：党中央、国务院和省委、省政府的关怀重视、大力支持，是加快赶超的根本前提。只有以强烈的政治责任感和使命感，化鼓励为动力、化鞭策为行动、化支持为成效，才能在上级的关心支持中实现更快发展，才能在加快发展中获得更大支持。核心党委坚强有力、四套班子共识共为，是加快赶超的关键所在。只有始终坚持在核心党委的领导下，几套班子干事业一条心、抓工作一个调、谋发展一盘棋，才能在和谐中凝聚更大合力、在共为中求得更大作为。主攻项目、加大投入，是加快赶超的强力引擎。只有坚持以项目主导发展、以投入拉动发展，围绕强工兴城的战略举措，心无旁骛、义无反顾地抓下去，三年翻番才有依托，五年进位才有支撑。科学谋划、精心操作，是加快赶超的有效手段。只有善于发现价值、利用资源，准确判断、化危为机，积极担当、破解难题，以科学的机制、到位的操作为“金钥匙”，不断解决前进过程中遇到的困难和问题，才能把赶超的步子迈得更大，把赶超的成效实现得更好。依靠群众、合力攻坚，是加快赶超的力量之源。只有紧紧团结和依靠全市人民，把赶超的强大合力凝聚在发展上、落实在工作中、体现在为民上，形成各条战线、各个地方、各个部门、各方群众“合心、合力、合成”的和谐局面，才能确保各项工作顺利推进。对党负责、为民服务，是做好政府工作的基本准则。只有坚守人民政府党领导、人民政府为人民的理念，坚定一切为了九江事业的发展、一切为了九江人民幸福的追求，坚持“两区互动、强工兴城”的战略不动摇，才能把九江人民的利益维护好，把九江奋起赶超的目标实现好。

各位代表，成绩已成过往，不可陶醉停歇；赶超引领未来，仍需奋力开拓。我们清醒地认识到：国际金融危机的影响仍然存在，赶超发展依然面临诸多挑战；尤其是兄弟地市竞相发展，态势逼人，要争得九江应有地位，任务十分艰巨；我市经济总量不大、实力不强，加快推进新型工业化和城镇化、壮大县域经济，显得尤为紧迫；群众就业、住房、增收等方面还存在较大压力，改善民生依然面临较重任务；在推进改革发展的过程中，影响安全稳定的因素还不少，机关效能还有待进一步提升，优化环境依然需要加大力度。对此，我们将冷静对待，深入研究，重点突破，科学破解。

　　二、2010年工作安排

　　2010年是实施“十一五”规划的最后一年，也是我市实施赶超战略、争得应有地位更为关键的一年。尽管宏观经济形势复杂，但我们也拥有诸多有利条件，加快发展的机遇更多；尽管区域经济竞争激烈，但我们已开创良好的赶超势头，加快进位的基础更牢；尽管前进过程中会有困难和压力，但我们在市委的坚强领导下，已形成强大的工作合力，加快赶超的信心更足。我们将咬住目标，精心组织，扎实工作，奋起赶超。

今年的政府工作，将在省委、省政府和市委的坚强领导下，以科学发展观为指导，紧紧抓住国家推进鄱阳湖生态经济区建设的战略机遇，加大强工力度，保持兴城势头，扩大投资规模，统筹各项工作，努力实现“双千双百”，乘势加快赶超步伐。综合考虑各方因素，全市经济社会发展的主要预期目标是：生产总值增长13%，完成970亿元，力争突破1000亿元；全社会固定资产投资增长40%，力争增长50%以上，突破1000亿元；财政总收入增长15%，力争增长25%以上，突破100亿元；整合各级投入25亿元以上，认真办好为民实事100件；社会消费品零售总额增长18%；居民消费价格总水平涨幅控制在4%以内；城镇居民人均可支配收入和农民人均纯收入增长8%以上；万元GDP能耗和化学需氧量、二氧化硫排放量控制在省政府下达指标之内；人口自然增长率控制在8.03‰以内。

实现上述目标，我们必须始终坚持增速度、扩总量这个根本不动摇，一手抓存量扩张，一手抓增量培育，不断扩大经济总量，以更快的速度实现赶超，以更大的跨度实现进位；必须始终坚持强工兴城这个重点不动摇，以新型工业化支撑新型城镇化，以适度超前的城镇化带动相对滞后的工业化，实现工业化和城镇化互动共进；必须始终坚持项目建设这个抓手不动摇，把抓项目、扩投资作为赶超发展的核心举措，多上快上项目，上大上好项目；必须始终坚持高效操作的机制不动摇，继续实行“六个一”、“六张作业单”等有效机制，以责任加压、以奖惩激励、以督查促进、以结果倒逼，确保各项工作高效推进。主要抓好以下七个方面的工作：

（一）继续加大强工力度，举全市之力决战工业2000亿。围绕“主攻园区，崛起两带（沿长江、昌九），三年实现主营业务收入2000亿”的目标，举全市之力决战工业，确保完成规模以上工业增加值增长20%以上，主营业务收入增长25 %以上，利税总额增长15%以上。

加大主攻园区力度，力促园区成为工业主战场。一是加强园区基础设施建设。各县（市、区）对工业园区基础设施投入每年不少于1亿元，九江开发区和共青城开发区基础设施投入每年增长30%以上；加快港口码头、沿江快速通道、沿江板块铁路专用线建设，启动城西保税港区申报前期工作，提高水陆双向物流能力；引导企业和民资参与园区基础设施建设，推进标准厂房和多层厂房建设，确定园区项目投资强度“底线”，提高园区集约发展水平。二是推进园区体制机制创新。对工业园区进行绩效考核，探索弹性用人机制，鼓励园区吸纳优秀专业技术人才和管理人才。三是完善园区服务和保障功能。搞好新市民公寓等生活配套设施建设，为园区务工人员提供有效生活保障，为项目落户、企业发展创造条件。四是提高园区承载力和吸附力。围绕“昌九四组团、沿江六板块、全市十三个园区”，推动园区产业集聚，打造一批有产业特色的专业工业园。力争新增2-3个主营业务收入过百亿的园区，九江经济开发区主营业务收入达到200亿元，共青城开发区、湖口金砂湾工业园、瑞昌工业园、城东港区、永修云山经济开发区主营业务收入增长40%以上，在全省位次前移。

加大龙头培育力度，力促产业集群加快形成。一是依托央企形成产业集聚。以中石化、中建材、中电投、国电集团等为重点，力争有合作基础的增资扩能，延展链条，配套集成；有合作意向的加强跟踪，落户项目，落实投资；力争更多的关联企业融入央企产业链条。加速推进彭泽核电、九江电厂四期、九江石化炼化一体化项目建设；鼓励现有央企投资企业通过技术改造、新产品开发，尽快做大做强。二是依托骨干企业形成产业集聚。继续推进石油化工、冶金建材、纺织服装、电力能源、汽车船舶等产业做大做强，通过技改扩能，做大九钢、龙达化纤、巨石玻纤、诺贝尔陶瓷、亚东水泥等企业规模；通过前后配套，做长汽车、造船、有机硅等产业链条；通过自主创新，加快培育一批拳头产品和龙头企业，逐步形成1000万吨钢材、1000万吨原油加工、2000万吨水泥、150万载重吨造船的产业规模，挺起工业经济“脊梁”。三是依托高新技术企业形成产业集聚。以武宁绿色照明高新技术产业园、旭阳雷迪500兆瓦多晶硅等项目为重点，支持太阳能光伏、新型材料、电子信息、生物科技、绿色能源等新兴产业加快发展。四是依托矿产资源形成产业集聚。抓住国家进行资源税改革的机遇，充分发挥矿产资源优势，大力规范矿产资源开发秩序，积极整合矿业资源，杜绝无序勘探、低端采选和初级原料输出。加强与江铜、中国五矿、中节能等企业集团的合作，使资源向龙头企业、优势企业集中，鼓励深度开发和精深加工，推进江铜铅锌冶炼、城门山二期、大唐氟化工等项目加快建设，推进香炉山钨业等矿业企业加快发展，形成以铜、钨、铅锌、硅、萤石等为主的矿业产业集群。

加大企业扶持力度，力促大企业做强、小企业做优。坚持既要“顶天立地”，又要“铺天盖地”，既抓拉动力强的重大项目，又抓成长性好的中小企业。一是对符合国家产业政策尤其是十大产业振兴规划的企业，帮助向上争取政策、资金和项目，引导企业建立研发机构和技术中心，鼓励企业实施名牌发展战略，争创中国驰名商标和国家级名牌，提高产品科技含量和附加值，并在用地、用工等方面给予帮扶。二是对列入省产业经济“十百千亿工程”和十大优势高新技术产业发展规划的企业，在生产要素上重点保障，在推进过程中重点调度，积极帮助企业与国家重大项目主动对接，开拓市场、抢抓订单。三是对创新创业的中小企业，全面落实 “五缓四减三补贴”措施，减轻就业型、成长型困难企业税费负担；积极推进政银企合作，加强项目融资对接，完善信用担保机制，简化担保审批程序，尽力满足企业资金需求。

加大国企改革力度，力促国企靠大联强焕发生机。一是资产清理到位。全面清理改制企业资产，利用市国有资产经营公司和市土地储备中心平台，统筹归集处置好企业资产。二是资产盘活到位。进一步盘活国有存量资产，适时处置资产、偿还债务，建立改制融资债务有序偿还机制，放大资产处置收益。三是产权改革到位。因地制宜采取战略投资者收购、靠大联强、股权置换、破产重组、整体转让等形式推进产权改革，盘活改制企业。继续推进维科印染、九针、德福电子、江氧、喜敦啤酒等企业退城进园。四是职工安置到位。做好职工身份置换的后续工作，推进改制企业的社会管理和组织工作，千方百计促进改制企业职工实现再就业。

加大考核激励力度，力促全市上下形成强工合力。一是加大领导力度，成立决战工业领导小组，进一步充实工业战线力量。二是加大考核力度，落实市级领导挂点帮扶重点企业责任，建立健全重大工业项目推进机制，推行“一名领导、一套班子、一事一议”的服务机制和“一周一督查，一月一调度，一季一通报，半年一考核，年终总评比”的督查考核机制，把工业发展情况和各单位服务企业情况，作为各地各部门和责任领导年终考核的重要内容。三是加大奖励力度，市财政每年安排1000万元专项奖励资金，奖励为全市工业发展作出突出贡献的工业企业及园区。四是加大扶持力度，市财政每年安排资金1000万元，建立企业发展扶持专项资金，重点支持企业技术改造、节能改造、技术创新。

（二）继续保持兴城势头，以强攻之势大力推进新型城市化。按照市本级两年完成100亿、五年完成200亿元投入的目标，以建设八里湖生态新区为主战场，逐步拓展城西港区、城东港区、十里片区，在尽快实现城区人口过百万的基础上，加快构建“大九江”格局。

注重规划引领。启动编制“大九江”发展战略规划，统筹搞好各空间板块之间的规划对接、功能设施对接和产业对接，推进“四县（市）一山”与中心城区相向发展。分片区修编完善中心城区控制性详规。按照“三个三分之一”的原则，完成八里湖东岸及濂溪大道等区域修建性详规编制和生态景观设计。策应鄱阳湖生态经济区建设，启动编制环湖城镇规划和生态规划。维护规划的严肃性和权威性，完善规划审批公示监督制度，健全公众参与机制。

加快项目建设。坚持“六个一”的机制，继续发扬"5+2"、“白+黑”的精神，高效推进城建项目。坚持基础设施和公共服务设施“两个优先”，着力推动以八里湖新区为重点的城建工程，进一步拉开新区框架，完善新区功能，做优新区环境。加快完成一批：完成九江火车站改造、九码快速通道、濂溪大道、十里河综合整治、八里湖环湖一路、环湖三路、八里湖大道、体育中心、新区学校、新区医院一期、中医院南院、南山路、兴城大道及体育中心配套路网、浔阳东路地下人防工程、庐山旅游直升机、赛城湖公园、鹤问湖污水处理厂配套管网、第二生活垃圾处理场等工程。适时启动一批：开工建设八里湖跨湖大桥、长虹东大道、滨江西大道、八里湖生态新区东片区及浔南片区路网、大中路历史街区改造等工程，加大路面维修养护力度，着力改善公交车通行条件，启动博物馆（规划展示馆）、文化艺术中心等工程的前期准备工作。改造提升一批：启动实施中心城区24万平方米棚户区（城市低洼危旧房住宅区）改造，完成浔阳东路、滨江路、长江大道等道路沥青路面改造，完善“两湖”周边景点布局，完成百条小街小巷改造，改善老城区人居环境。通过运作城市土地、城市资本、城市资源筹资融资，确保举债有度、用债有方、还债有源，力争全年新增50亿元城建投入。积极创建国家园林城市和省生态园林城市，启动实施南湖公园、十里河景观公园、八里湖滨湖公园、奥林匹克体育公园、南山公园、龙开故道景观公园、天花井森林公园、好汉坡登山公园等十大城市公园的新建和改造提升，逐步建设完善十个精品绿地，开展十大园林绿化示范小区、十个园林绿化示范单位的创建活动，提高园林绿化和管养水平。

提升管理水平。坚持以人为本，健全专业化城市公共服务平台，畅通市民诉求渠道，继续做好全国无障碍城市创建工作，提升城市管理人性化水平；巩固“两声三乱”整治成果，加大市容市貌、环境卫生、交通秩序专项整治力度，保持城区大街小巷市容整洁、畅通有序，积极推行“和谐城管”，提升城市管理规范化水平；从市政设施、建筑工地、小区物业、市场秩序等方面着手，健全城市管理网络，提升城市管理常态化水平；综合运用现代化信息技术，加强对城市基础设施和管理活动的实时监测与动态控制，提升城市管理信息化水平；深化城管体制改革，有序推进城市公用事业建设和市容环卫公共服务市场化，提升城市管理市场化水平。

繁荣城市经济。做大城市人口基数，扩大城市消费需求，提高城市居民购买力，通过集聚人气繁荣城市经济。按照“把新区建设成为商业、文化、旅游集散和服务接待中心”的目标，推进星级宾馆、高档写字楼、美食街区等基础设施和服务功能设施建设，逐步形成布局合理、功能配套、设施齐全的社会综合服务能力。加快五金电器市场、维科纺织城、小商品综合批发市场和城东水产品批发市场等项目建设，启动京九批发市场、华东建材市场改造，积极发展总部经济、楼宇经济、现代物流、金融保险、商业连锁、电子商务、文化创意等服务业态，提升现代服务业发展水平。

（三）继续扩大投资规模，以主攻项目实现全年固投1000亿。坚持把项目建设放在重于一切的位置，进一步扩大投资需求，力争全年固定资产投资增幅达50%以上，突破1000亿元。

招商引资上项目扩固投。进一步扩大对外开放，加大招商引资力度，加强与中国500强、中国“五会”、中青企协等企业和组织的对接，不断提升开放型经济发展水平。抓住港台和沿海发达地区产业加速内迁的时机，大力开展产业招商，力争引进亿元以上重大项目100个以上，实际利用外资增长10％以上；实际引进市外5000万元以上工业项目资金增长20％以上。大力实施“走出去”战略，开展对外经济合作，扩大外贸出口规模，力争全市外贸出口增长20％以上。

向上争资上项目扩固投。深入研究国家政策，准确把握国家在中部地区投资重点和趋向，积极策应《促进中部地区崛起规划》和“十二五”规划编制，以基础设施建设为重点，扎实做好各类项目的深度谋划、编报和争取工作。加快九池城际铁路、咸宜吉铁路、九岳铁路等项目的对接、跟进、配套工作，争取挤进国家和省投资计划“笼子”；加快推进并力争开工都昌蔡岭至马回岭加密高速；开工建设武九客专、九江绕城高速、九景衢铁路；建设完成京九线电气化改造、武九线电气化改造、昌九城际铁路、九瑞高速、彭湖高速、柯龙线改造；加速推进马回岭500千伏输变电站等十大电网、长江二桥、中石油湖口油库等一批重大项目建设。

激活民资上项目扩固投。进一步激活各类创业主体，支持经营管理和科技人员领头创业，吸引外出务工经商人员返乡创业，大力扶持下岗失业人员自主创业，鼓励高校毕业生自立创业，推动广大农民致富创业，引进市外人才和归国留学人员来浔创业。按照非禁即入原则，放开市场准入，降低创业门槛，减少创业成本。积极建设一批创业园、创业中心、创业社区，新建10个小企业创业基地，优先保障创业用地。搞好创业免费培训，优化创业信息服务，定期发布创业指导项目。激发民间投资创业热情，变民间存量资本为现实投入，力争民营资本投资占全社会投资70%以上。

创新机制抓融资扩固投。进一步发挥现代金融在推进赶超中的重要作用，加强与商业金融机构的战略合作，吸引更多银行来浔布点，加大信贷投放量；加快政府性资产投融资平台建设，整合有效资产，盘活有效资源，形成项目建设有资金保障、资金来源有资产保障、资产资源由金融盘活的良性循环。探索成立股权多元化的担保公司，有效增强直接融资能力，力争新组建小额贷款公司3-5家、村镇银行2-3家；积极放大九江城投债券投资效益；大力支持各家银行在九江加快发展，支持九江银行做大做强；支持财兴卫浴、九江银行、银星造船、美庐乳业、联盛集团等企业上市融资。

（四）继续加快发展县域经济，增强县域经济对赶超发展的支撑力。以强化“三农”基础、加大帮扶力度、推进城镇建设为重点，加快发展县域经济。力争全年9个县（市、区）财政收入过5亿元，其中过9亿元的1个、过8亿元的2个、过7亿元的3个。

着力抓“三农”，夯实县域经济的发展基础。坚持以工业化发展农业，以城镇化带动农村，以产业化致富农民。全面落实各项强农惠农政策，稳定粮食种植面积，努力提高粮食单产和品质，确保全市粮食总产稳定在29亿斤，力争农民收入增长8%以上。加快推进农业产业化步伐，组织实施 “十百千万”工程，力争每个县区新引进3000万元以上农业项目2-3个，亿元以上项目1-2个。实施"4545"工程，力争规模以上农业企业销售收入增长20%以上。加强农村基础设施建设，全面完成专项规划内81座中小水库除险加固任务，新增标准农田8万亩、有效灌溉面积8万亩、旱涝保收面积5万亩；以村庄整治为切入点，建好1100个新农村建设点。深入推进农场税费改革和林权配套改革，合法有序推进农村土地流转；全面启动农技推广体系改革，建设乡镇农业技术综合站100个，建成市级农产品检验检测中心，启动县级农产品检验检测中心建设，推进农村信息化工程，提升服务“三农”水平。健全以社会养老保险、医疗保险、被征地农民基本生活保障、最低生活保障为主的“四道保障线”，不断提高农民的保障性收入。

着力抓帮扶，激活县域经济的发展动力。进一步加大放权力度，扩大下放审批事项范围，畅通县区项目建设的报批渠道，增强县域发展自主权。进一步加大支持力度，全力服务基层，把县区难题当作市本级的难题，把县区需求当作市本级的需求，继续采取现场办公等有效方式帮助县区攻坚破难，加快发展。进一步加大助推力度，对县区进行分类指导，支持沿江、沿湖、沿路和山区县发挥各自优势，发展壮大支柱产业，做强县域特色经济板块。进一步加大引导力度，建立市与县、县与县联动发展机制，协调推进设施共建共用、城乡协同发展、产业联合打造，有序引导条件受限的县按“成本分担，利益分成”原则到九江开发区建园区，发展“飞地”经济。进一步加大激励力度，健全县区项目调度督查考核机制，继续实行县域经济半年度现场推进会、年终巡回检查制度和奖励制度，促进县域经济竞相发展。

着力抓城镇，增强县域经济的发展活力。加大对县城和重点集镇建设的指导支持力度，进一步推进城镇规划修编，加快城镇控制性详规编制。把小城镇建设与培育特色优势产业结合起来，与减少“空心村”结合起来，与服务“三农”结合起来，与完善农村社会保障体系结合起来，与加快农村富余劳动力转移就业结合起来。以实施“百镇示范工程”为契机，以港口街镇、新港镇等滨湖、沿江和沿路区域为重点，着力打造一批中心集镇，使之成为辐射和带动农村发展的消费中心、生产要素集散中心、“三农”服务中心、社会保障和公共服务中心。围绕做强实力、培育产业、完善功能、提升品位，积极开展“扩权强镇”试点工作，扶持一批重点乡镇，不断壮大乡镇经济，着力打造一批在区域有影响力、县域有支撑力、领域有带动力的特色乡镇。推进户籍制度改革，放宽户籍限制，推动农村人口加快向城镇转移。

（五）加强生态建设，努力争当鄱阳湖生态经济区建设的排头兵。抓住国家推进鄱阳湖生态经济区建设的战略机遇，充分发挥生态优势和主体作用，精心组织规划实施，突出生态特色，提升产业层级，不断探索生态经济化、经济生态化、生态经济效益化的新路。

积极策应鄱阳湖生态经济区战略，加强生态保护。配合做好鄱阳湖生态经济区重点领域规划编制，抓紧启动市县乡配套规划编制及产业配套政策制定工作。严格项目环保准入，从源头上遏制环境污染；继续开展“修河环保行”等专项整治行动，加强沿河、沿江和环湖等环境敏感区的生态保护；强力推进污染减排，完善管网配套，推行雨污分流，提高城镇污水处理厂、企业重大减排设施的投运率，确保达标排放；加强环境监测监察，提升环境监管能力，维护生态环境安全。继续坚定不移推进鄱阳湖采砂统一管理。

积极实施鄱阳湖生态经济区规划，建设生态工程。精心打造“十里香樟走廊、百里昌九绿带、千里绿色通道、万亩精品园林”，构筑生态九江的绿色屏障。继续开展生态县、环境优美乡镇创建工作，大力推进以农村生活垃圾无害化处理为主的农村清洁工程，着力抓好农村生态建设。配合做好彭泽核电一期工程技术设备试验和鄱阳湖生态水利枢纽工程前期工作，加强湿地候鸟、鱼类洄游、江湖关系等专题研究。抓好新型能源工程建设，加快推进老爷庙风电、长能风电设备、华航光电、中辉特光伏等项目建设，启动核电配套产业园的前期工作，促进清洁能源规模化开发，加快打造中部绿色能源基地。

积极推进经济增长方式转变，发展生态经济。加快发展低碳与生态经济，逐步构建以生态农业、新型工业和生态旅游为支撑的环境友好型产业体系。大力发展生态农业，依托农副产品资源，抓好一批生态农业项目，争创无公害、绿色、有机食品品牌，提高生态农业经济效益。大力发展新型工业，抓好生态园区建设，推进节能和循环经济加快发展，加大对余热发电、废物再利用项目支持力度，扶持武宁节能灯产业做大规模，加快挪宝电器、卢氏LED等节能项目建设，努力打造一批新兴产业基地和特色经济板块。进一步挖掘各种旅游形态的市场潜能，积极融入环鄱阳湖五彩精华旅游圈和赣鄱文化山水旅游黄金线路，大力发展生态旅游、乡村旅游、文化旅游和度假休闲旅游；加快推进庐山北门下迁、庐山西海旅游高速、西海国际艺术园、中信集团西海开发等项目，提高景区档次，提升产业层次，推进旅游资源大市向旅游经济强市转变。

积极支持共青城超常规崛起，打造生态样板。坚持现代化、国际化、低碳化的发展模式，充分发挥共青城的独特优势，认真落实好支持共青城跨越式崛起的政策措施，加快推进中国青年创业基地、中芬数字生态城、中加绿色经贸合作开发区、中航文化产业园和鄱阳湖物理模型试验研究基地建设，着力推动共青城-德安相向发展、融为一体，加速将注意力优势转化为现实的经济优势，全力把共青城打造成经济文明、社会文明和生态文明有机统一的样板，打造成鄱阳湖生态经济区的亮点，打造成国际合作的示范区，打造成加快九江发展、实现江西崛起的一个重要增长点。

（六）加强社会建设，大力推进以改善民生为重点的社会事业全面发展。坚持一手抓发展、一手托民生，加大力度保民生、保稳定、促统筹，推动各项社会事业全面进步。

继续实施民生工程。本着量力而行、尽力而为、可持续实施的原则，在全面完成省政府下达民生工程八大项54件指标任务的前提下，通过广泛征集群众意见，结合实际，再新增五大项46件惠民实事，总共达到100件（详见附表）。为确保这些民生实事顺利推进，全面完成，惠及民众，在实施过程中，一是更加强化宗旨观念，全心全意为人民服务、为人民办事，尽最大努力将这些民生实事办好，尽最大能力将群众幸福指数提高；二是更加强化责任意识，继续将民生工程列入政府工作“六张作业单”，年初分工，过程跟踪，年底交帐。同时，由于民生工程的受益群体主要在基层、在农村，市政府将与各地签订责任书，层层分解任务，加强协调调度，提高审批效率，力促早动手、早建设、早兑现；三是更加强化资金保障，坚持做到“四个倾斜”，加大民生工程资金筹集力度，压缩一般性公用经费，确保资金拨付及时足额到位，加强资金管理使用监督检查，提高资金使用效益；四是更加强化督查考核，加强分类指导，改进督查方法，对实施情况进行年度考核评比，严格兑现奖惩，确保民生工程平衡、有序实施；五是更加注重实施效果，组织到位，操作到位，兑现到位，结果公诸于众，做到取信于民，确保各项实事快推进、人民群众得实惠。

切实维护社会稳定。全面落实安全生产“一岗双责”责任制，深入开展安全生产隐患排查整治，保障道路交通、危化物品、食品药品、森林防火、城市消防等重点领域安全，有效防控各类安全事故发生。继续巩固完善和贯彻落实好领导包案、领导接待日、干部下访等制度，依法依规解决群众合理诉求，确保“四个不发生”。切实维护国家安全，严密防范和打击境内外敌对势力的破坏活动。积极排查化解矛盾纠纷，创新社会组织服务管理模式，有针对性地组织开展“打黑除恶治霸”等专项打击行动，逐步推进“天网工程”和信息化建设，大力整治治安乱点，切实增强群众安全感。

协调发展各项事业。进一步落实教育优先发展战略，继续改善农村中小学办学条件，加强农村师资队伍建设，促进城乡教育均衡发展；支持高等教育发展，充分发挥高等教育服务地方经济发展的功能和潜力；协调发展职业教育、特殊教育、幼儿教育；加快教育现代信息技术工程建设；加强未成年人思想道德教育；积极创建“和谐平安校园”；做好二类城市语言文字评估验收工作。提升科技创新能力，加快科技成果转化，提高科技对经济的贡献率，力争新认定高新技术企业增加一倍，高新技术产业产值增长20%以上；加强科技对接，促进产学研合作，推进科技入园、科技进村入户工程，力争转化科技成果100项以上；加大高层次人才引进力度。全面推进新医改，进一步健全公共卫生服务和疾病预防控制体系，完善城市社区和农村卫生服务网络；推广残疾人社区康复工程。深入挖掘文化资源，制定发展规划，出台支持政策，加快文化产业发展；推进文化体制改革和机制创新；开展全民阅读活动，创新农村文化专项活动，弘扬传统文化，繁荣文化市场，创建学习型城市。认真贯彻《全民健身条例》，广泛开展全民健身运动，积极备战省运会。鼓励社会资本投资兴办福利机构，依托社区发展居家养老服务，逐步提高社会福利服务水平。完善应急管理体系，增强突发公共卫生事件应急能力。构建慈善组织网络，实施慈善救助项目。切实推进殡葬改革。着力抓好人口和计划生育工作。认真做好第六次全国人口普查工作。抓紧编制“十二五”规划。强化统计、审计、档案、编制、信息化工作，提高经济社会管理水平。加强对台、外事侨务和民族宗教工作，促进友好交流。支持工会、共青团、妇联等群团组织发挥作用。开展国防教育，启动新一轮双拥模范城创建工作。

（七）加强效能建设，不断提升各级政府推进赶超发展的实现力。围绕打造“对党负责、为民服务、高效务实、清正廉洁”政府团队的目标，以深入开展“创业服务年”活动为载体，不断加强政府自身建设，不断提高推进赶超发展的实现力。

努力把上级的要求实现好。进一步加强学习，不断提升复杂环境下驾驭经济工作的水平和依法行政的能力。在市委的坚强领导下，自觉接受人大依法监督和政协民主监督，认真办理人大议案、代表建议和政协提案，主动听取各民主党派、工商联、无党派人士和人民团体的意见与建议，自觉接受人民群众和新闻媒体对政府工作的监督。切实强化行政执法监督，严格落实行政执法责任制和责任追究制，全面规范行政处罚自由裁量权，严肃整治行政不作为、慢作为和乱作为，坚决查处破坏发展环境的人和事。建立健全公众参与、专家论证和政府决定相结合的行政决策机制，提高决策的科学化和民主化水平。继续围绕省考核体系，突出重点，兼顾全面，努力争先进位。

努力把发展的需求实现好。继续健全完善政府工作“六张作业单”的运行机制，把工作任务细化、量化、责任化，使政府各项工作分工更加明确，责任更加强化，执行更加高效。深化行政管理体制改革和机构改革，理顺部门职能分工，营造“争创一流工作，争做优秀部门”的良好氛围，打造团结、务实、高效的政府团队。坚持以担当的姿态，努力用合法高效的操作突破体制机制瓶颈，用科学精细的操作推进重大项目建设，用务实创新的操作赢得改革发展先机，用有情融通的操作保障群众切身利益，不断破解制约赶超的难题，不断铺平加快赶超的道路。

努力把群众的盼求实现好。进一步掌握群众所盼，畅通诉求渠道，全面完善“民声直通车”办理平台，继续强化分管负责、部门落实的办理机制，为人民办事，让人民满意。全面落实党风廉政建设责任制，健全惩防体系，从源头上治理和预防腐败。加强对权力运行的监督约束，强化行政监察和审计监督，全面建设网上审批和电子监察系统，继续推进“廉政阳光工程”建设，深入推行财务财政票据电子化和公务卡制度，扎实开展对工程建设、土地出让、产权交易、政府采购等重点领域的突出问题专项整治工作。严格执行领导干部廉洁自律规定，发扬艰苦奋斗、勤俭节约传统，杜绝挥霍浪费，集中更多财力物力促发展、保民生。

各位代表，科学发展、奋起赶超，是众望所归；三年翻番、五年进位，是目标所指；两区互动、强工兴城，是战略所向。让我们在省委、省政府和市委的坚强领导下，高举中国特色社会主义伟大旗帜，全面贯彻落实科学发展观，凝心聚力，扎实工作，乘胜追赶，乘势而超，为争得九江应有地位而努力奋斗！
